# Cysteine Aminotransferase (CAT): A Pivotal Sponsor in Metabolic Remodeling and an Ally of 3-Mercaptopyruvate Sulfurtransferase (MST) in Cancer

**DOI:** 10.3390/molecules25173984

**Published:** 2020-09-01

**Authors:** Ana Hipólito, Sofia C. Nunes, João B. Vicente, Jacinta Serpa

**Affiliations:** 1 CEDOC, Chronic Diseases Research Centre, NOVA Medical School|Faculty of Medical Sciences, University NOVA of Lisbon, Campus dos Mártires da Pátria, 130, 1169-056 Lisbon, Portugal; ana.hipolito@nms.unl.pt (A.H.); asofianunes5@gmail.com (S.C.N.); 2 Institute of Oncology Francisco Gentil (IPOLFG), Rua Prof Lima Basto, 1099-023 Lisbon, Portugal; 3 Institute of Technology, Chemistry and Biology António Xavier (ITQB NOVA), Avenida da República (EAN), 2780-157 Oeiras, Portugal

**Keywords:** cysteine aminotransferase (CAT), 3-mercaptopyruvate sulfurtransferase (MST), cancer, metabolic remodeling

## Abstract

Metabolic remodeling is a critical skill of malignant cells, allowing their survival and spread. The metabolic dynamics and adaptation capacity of cancer cells allow them to escape from damaging stimuli, including breakage or cross-links in DNA strands and increased reactive oxygen species (ROS) levels, promoting resistance to currently available therapies, such as alkylating or oxidative agents. Therefore, it is essential to understand how metabolic pathways and the corresponding enzymatic systems can impact on tumor behavior. Cysteine aminotransferase (CAT) per se, as well as a component of the CAT: 3-mercaptopyruvate sulfurtransferase (MST) axis, is pivotal for this metabolic rewiring, constituting a central mechanism in amino acid metabolism and fulfilling the metabolic needs of cancer cells, thereby supplying other different pathways. In this review, we explore the current state-of-art on CAT function and its role on cancer cell metabolic rewiring as MST partner, and its relevance in cancer cells’ fitness.

## 1. Introduction

Cancer metabolism is one of the oldest studied research topics in oncobiology, based on the principle that metabolic activities are altered in cancer cells relatively to normal cells, and that these alterations support the acquisition and maintenance of malignant properties. Accordingly, and since various altered metabolic features are observed across many types of cancer cells, reprogrammed metabolism is now considered a hallmark of cancer [1,2]. In fact, metabolic reprograming in cancer implies a general switch in cellular demands that derive from its altered behavior and consequently requires cells to increase ATP production through alternative and/or additional pathways as compared to normal cells. The increased demand for ATP contributes to maintain cancer cells viability and supports malignant features, such as the improved proliferative activity and survival capacity.

A specific feature of certain cancer cells, also linked to altered metabolic features, is the intrinsic or acquired resistance to common chemotherapeutic oxidative and/or alkylating drugs, also designated as chemoresistance. Among different adaptive strategies adopted by chemoresistant cancer cells, chemoresistance has been associated with alterations in glutathione (GSH) and cysteine dynamics. We and others showed that, overall, cysteine flux in cancer cells and the expression and activity of enzymes involved in cysteine metabolism interfere with the response to chemotherapy by ultimately abrogating the effect of oxidative or alkylating drugs [3,4,5,6,7]. On the other hand, enzymes function is influenced by the oxidative stress. Thus, GSH and cysteine play a very important role not only in allowing the survival of cancer cells in adverse pro-oxidative conditions, such as radio- and chemotherapy, but also in the metabolic remodeling, conferring a deep reliance on thiols. The relevance of this topic in cellular and systemic cancer metabolism has been extensively reviewed and explored in different cancer models [8,9,10,11,12].

The hydrogen sulfide (H_2_S)-generating enzymatic system composed of cysteine aminotransferase (CAT, EC 2.6.1.3, also known as aspartate aminotransferase, AST, or glutamate transaminase, GOT), and 3-mercaptopyruvate sulfurtransferase (MST, EC 2.8.1.2), is known to be implicated in the catabolism of cysteine [13], but its role is far from being completely explored in cancer. Therefore, this review aims to present a comprehensive review of the available literature regarding the stand-alone function of CAT in amino acids metabolism, and as component of the CAT:MST metabolic axis, in the context of cancer.

## 2. Cysteine Aminotransferase Structure and Localization

Two isoforms of CAT, classified according to their cellular localization as cytoplasmic (cCAT) and mitochondrial CAT (mCAT), are encoded respectively by *GOT1* (glutamic-oxaloacetic transaminase 1) and *GOT2* (glutamic-oxaloacetic transaminase 2) [14], which are extremely well conserved across species [15,16]. CAT is a homodimeric pyridoxal phosphate (PLP)-dependent aminotransferase [17,18], being considered the best studied PLP-dependent enzyme, given its easy large-scale purification and its stability [19,20]. The mCAT was in fact the first PLP-dependent enzyme to have its X-ray structure determined [21], leading to key insights on its catalytic mechanism, holding to this day [19]. Each cCAT monomer consists of two domains: A small domain composed of four α-helices and three β-strands, and a large domain comprising a 7-stranded β-sheet and several short α-helices. Protein dimerization is ensured by the large domains, with two PLP-binding sites which are stabilized by the surrounding residues [20]. PLP has a dual physiological effect: While it is vital for normal cellular metabolism, excessive levels of free PLP can non-specifically and covalently bind to thiol and amino groups [22,23]. Indeed, spontaneous formation of hydrogen sulfide (H_2_S) by free PLP and cysteine has been reported [24]. Mutations in cCAT impact on its structure and prevent effective PLP binding by a dimer interface misalignment, which can lead to its dissociation and PLP release [20].

Firstly isolated from rat liver [25,26,27], both mCAT and cCAT were primarily studied within the context of pathologies associated with defects in the cysteine degradation pathway [28,29]. Overall CAT activity is higher in heart and liver, but it is also detected in kidney, brain, and skeletal muscle [17,18,30]. Regarding cysteine metabolism, CAT is generally referred to in association with MST, the focus of most studies. Meister (1953) and Wood and Fiedler (1953) first described the cysteine-catabolizing enzyme MST in rat liver, and it was further found to be distributed and well conserved throughout prokaryotic and eukaryotic organisms [31,32,33,34,35]. MST is a Zn-dependent enzyme, member of the rhodanese-like sulfurtransferase family, containing two rhodanese-like domains and existing in a monomer-dimer equilibrium, the first being the active form [13,36,37]. MST presents a broad distribution in most mammalian tissues, although its expression is tissue-specifically regulated, being at high levels in kidney, liver, brain, testes, large intestine, and endocrine organs [34,36,38]. Interestingly, an alternative function has been proposed for MST which may be relevant in the context of cancer cell biology. Indeed, Frasdorf and co-workers described MST as a tRNA thiouridine modification protein (TUM-1), being implicated in the thiolation of cytosolic tRNA, and identified two isoforms of this protein in human cell lines, presenting different subcellular localizations: TUM1-Iso1 was identified exclusively in the cytosol; and TUM1-Iso2 was found to be expressed both in the cytosol and the mitochondria [37]. Interestingly, both isoforms are reported as exhibiting similar kinetic parameters, including protein stability, pH dependence and catalytic constants in the presence of non-physiological acceptors, as cyanide and DTT [37,39]. As mentioned, studies have clarified the subcellular localization of both CAT and MST in cytosol (cCAT; cMST) and mitochondria (mCAT; mMST). However, the CAT:MST axis may be more relevant in the mitochondria, since this is the site where cysteine is preferentially found [38].

### 2.1. Regulation of GOT1 and GOT2 Genes Expression in Cancer

Several genetic variants were reported for both genes encoding CAT enzymes (*GOT1* and *GOT2*) [40]. According to available RNA-seq data, high expression levels of cCAT-encoding *GOT1* and mCAT-encoding *GOT2* are detected in several cancer types, including liver, colorectal, prostate, head and neck, cervical, stomach, endometrial carcinomas, and melanoma. Additionally, high *GOT1* expression occurs in renal cancer and urothelial carcinomas [41].

Hong and colleagues showed that the breast cancer susceptibility gene 1 (*BRCA1*)-encoded protein BRCA1 formed a co-repressor complex with Zinc finger and BRCA1-interacting protein with KRAB domain-1 (ZBRK1) on the *GOT2* promoter via the ZBRK1 recognition element, suppressing its transcription [42]. Overexpression of mCAT is frequently reported in breast cancer. Furthermore, *BRCA1* deficiency accelerated tumor growth along with *GOT2* upregulation by its transcriptional activation (or derepression), which indicates that mCAT could act as a metabolic malignancy driver in the absence of BRCA1 or ZBRK1 in breast cancer. Interestingly, the authors also reported that mCAT-overexpressing breast cancer cells were more sensitive to methotrexate treatment, suggesting this enzyme as a possible marker towards personalized medicine, aiming to anticipate therapy resistance in these breast cancer patients [42]. However, BRCA1 loss-of-function may explain these results, possibly limiting the beneficial benchmarking of mCAT expression in patients with BRCA1 mutations. The somatic *BRCA1* mutations are not screened in breast cancer with unrecognized familial history, but they are estimated as being rather frequent in early and old onset patients of breast cancer [43]. Furthermore, in addition to breast cancer [42], mCAT overexpression was found in large B-cell lymphoma patients [44]. In B cells lymphomas, the pro-inflammatory pathways of cytokines and tumor necrosis factor (TNF) α play a role in the regulation of mCAT expression, since *GOT2* was described as being a direct target of signal transducer and activator of transcription–3 (STAT3) and p65 nuclear factor kappa B (NF-κB) regulation [44], the latter being, interestingly, a target of H_2_S-mediated persulfidation [45].

Zhang et al. explored the role of micro RNAs (miRNAs) in the regulation of *GOT1* (cCAT) expression and showed that miRNA-9 can down-regulate *GOT1* expression in melanoma cells [46]. Interestingly, this group further reported that *GOT1* down-regulation by miRNA-9 rescued cells from ferroptosis, an iron-dependent molecular mechanism associated to cell death. Ferroptosis is inhibited by the GSH-dependent glutathione peroxidase 4 (GPX4) activity, which is able to scavenge the iron-derived reactive oxygen species (ROS), and avoid lipids peroxidation that will induce membranes damage and cell death [47]. Because cysteine is a limiting component of GSH, cysteine levels control ferroptosis. It is thus likely that silencing CAT contributes to maintain cysteine available for ferroptosis inhibition [48]. Accordingly, Zhang et al. showed that suppression of miRNA-9 increased the sensitivity of melanoma cells to ferroptosis-inducing drugs [46]. Reinforcing these findings in cancer, Wang and colleagues showed that miRNA-9-5p inhibits the expression of cCAT in pancreatic cancer cells [49].

### 2.2. Regulation of Cytosolic and Mitochondrial CAT Activity in Cancer

Data is still scarce considering the relevance of CAT enzymes expression and activity in cancer; however, evidence suggests that their expression is highly dependent on cell metabolic states and stimuli from the tumor microenvironment (TME). The mCAT form was found to be abundantly expressed in normal head and neck tissue, but its reduced expression in tumor cells of patients with human papillomavirus (HPV)-positive head and neck squamous cell carcinoma was associated with improved overall survival (OS) [50]. Overexpression of mCAT was reported in breast cancer, particularly in triple negative breast cancer [42], and in lymphoma, with prognostic value by correlating with worse OS in large B-cell lymphoma patients [44]. cCAT expression status was further reported by Feld et al. as an independent prognostic marker of pancreatic ductal adenocarcinoma, allowing the stratification of patients in subgroups with a median OS variation of approximately 6 months, in which patients with cCAT-positive tumors presented a significantly longer OS and earlier stage of the disease [51].

Post-translational modifications (PTM) play important roles in the regulation of protein function, structure, and stability. Lysine acetylation is an evolutionarily conserved PTM that is known to participate in the regulation of a wide range of cellular processes, particularly in nuclear transcription and cytoplasmic metabolism [52,53,54]. mCAT can be acetylated in three lysines and its acetylation load is dependent on sirtuin-3 (Sit-3) function. When acetylated, mCAT is more active, which contributes to ensure the functioning of critical pathways to provide energy and support pancreatic cancer cells proliferation and tumor growth [53,54,55]. To our knowledge, no PTM of cCAT have yet been reported in relation to cancer.

### 2.3. Genetic Alterations in Cytosolic and Mitochondrial Cysteine Aminotransferase and Metabolic Consequences

Given the important detoxifying and catabolic functions performed by CAT by itself or within the CAT:MST enzymatic axis, it is expected that genetic alterations in these genes lead to a deregulated catalysis, accumulation of substrates, and depletion of reaction products, such as H_2_S and, ultimately, ATP. Next, we will review reported variants that correlate with CAT, given its wide importance.

*GOT1*, encoding cCAT, was found to be mutated (c.622C>G; p.Q208E) in familial macro-aspartate aminotransferase (macro-AST) patients, a benign condition characterized by elevated serum AST activity and associated to the presence of the macroenzyme form of cCAT (complex between cCAT and serum immunoglobulins IgA, IgG, or both). Bioinformatics analysis of this variant indicated that it probably does not affect the enzyme catalytic site. Although this is a rare variant and probably not the only factor causing familial macro-AST, this mutation can be associated to this pathology probably by inducing a stronger association between cCAT and serum components [56]. Moreover, another study reported a deletion in *GOT1* (c.1165_1167delAAC), which seemed to induce significantly lower AST activity, but an association with metabolic consequences was not found [57]. An interesting variant of *GOT2*, though not common, was found in pheochromocytomas and paragangliomas. Tumors bearing this variant (c.357A>T; p.E119D), presented significantly higher expression and activity of mCAT and high succinate/fumarate ratio, as well as a slight increment of α-ketoglutarate. Moreover, lymphocytes carrying this mutation, immortalized by Epstein-Barr virus, also exhibited significantly higher mCAT enzymatic activity, when compared to *GOT2*-wild type lymphoblastoid cell lines. Additionally, HeLa cells expressing this mutation, reproduced the effects seen in tumors regarding succinate/fumarate dynamics and α-ketoglutarate/citrate and aspartate/glutamate ratios, suggesting an activating role for this mutation that can promote an increased incorporation of α-ketoglutarate in the tricarboxylic acid (TCA) cycle and lead to the oncogenic accumulation of succinate [58].

The physiological relevance of mCAT is pointed by the fact that homozygous *GOT2*-knock-out mice and zebrafish are not viable, as showed by van Karnebeek and colleagues [59]. In the same study it was reported that *GOT2* (mCAT) bi-allelic mutations promote a mitochondriopathy through deficiencies in mCAT functioning. Despite showing residual mCAT enzymatic activity, affected patients present clinical evidence of this deficiency, including metabolic encephalopathy with epilepsy, progressive microcephaly, and several biochemical abnormalities [59]. Importantly, mutated mCAT promotes a dysfunctional malate-aspartate shuttle (MAS), which leads to a deregulation in the cellular NADH/NAD^+^ equilibrium, affecting NAD-dependent enzymes and pathways [59]. MAS relies on the functionality of two main operators, glutamate oxaloacetate transaminases (GOT, also known as CAT) and malate dehydrogenases (MDH), both present in the mitochondria and in the cytoplasm [55]. A decrease in serum levels of serine was also found in the patients studied by van Karnebeek and colleagues, which was probably correlated to this dysfunction, particularly with the increased NADH/NAD^+^ ratio, since no mutations were found in the serine biosynthesis-related genes that could explain this. Accordingly, the first step of de novo serine biosynthesis relies on the NAD^+^-dependent enzyme 3-phosphoglycerate dehydrogenase. Moreover, glycine production was also affected, which is consequently also related to the deficient MAS [59], since glycine biosynthesis depends on serine bioavailability. Additionally, deficient MAS also induced increased lactate serum levels [60].

Cells are highly dependent on mitochondrial aspartate production to fuel pathways such as the urea cycle. Mutated-GOT2-induced deficient MAS impairs mitochondrial synthesis of aspartate, leading to its decreased availability both in mitochondria and cytosol [59,61]. Sullivan et al. suggested aspartate as a limiting metabolic intermediate, promoting cancer growth arrest, but not with the same limiting potential across all cancer types, possibly depending on the process through which cells acquire aspartate or their metabolic gene expression [62]. This reinforces the idea that CAT may not be an appropriate therapeutic target, but rather could serve to highlight clues on tumor susceptibility and, eventually, support the prediction of therapy response.

## 3. CAT Role in Physiology and in Cancer

CAT’s substrate promiscuity makes it an interesting enzyme in different metabolic and (patho)physiological contexts and thus, it is denoted in the literature accordingly. Moreover, it interplays with several metabolic processes, mainly regarding amino acid metabolism. Despite being mostly studied regarding their role in cysteine metabolism, both cCAT and mCAT can additionally use other substrates like aspartate and alanine sulfinic acid, each additional function deserving to be separately explored [26,29].

### 3.1. CAT as an Aspartate Aminotransferase

The crosslink between CAT’s different metabolic pathways constitutes a complex metabolic map of reactions. CAT substrates can interfere with each other’s metabolism. As an example, high l-aspartate levels can competitively inhibit H_2_S production from l-cysteine through the CAT:MST system [25,26]. cCAT is a part of the MAS, and catalyzes the bidirectional interconversion of aspartate and α-ketoglutarate into oxaloacetate (OAA) and glutamate [63]. This reversible reaction occurs via two half-reactions in a “ping-pong” mechanism, the first comprising the reaction of the PLP moiety with l-aspartate, yielding pyridoxamine 5′-phosphate (PMP) and OAA; and the second consisting of the reverse of this half-reaction with α-ketoglutarate, restoring PLP and generating l-glutamate [19]. Once aspartate levels decrease, CAT can: (i) Reverse the flux to generate rather than consume aspartate (upon mitochondrial electron transfer chain dysfunction [19,64]), which is required for purine and pyrimidine synthesis; (ii) sustain the urea cycle by acting as a nitrogen donor [65]; and (iii) underlie the production of arginine as well as asparagine, by the catalysis of asparagine synthetase [66].

Most human cells are unable to effectively uptake aspartate given the low expression levels of aspartate/glutamate transporters and so, they rely on its intracellular synthesis by mCAT.

Within glutaminolysis, cCAT can use the aspartate produced by mCAT to produce OAA, which, besides fueling other pathways, can be converted into lactate through the OAA-malate-pyruvate axis, in normoxia. Additionally, in cancer cells, mCAT can convert glutamate to 2-oxoglutarate, which is a glutaminolysis intermediate. However, hypoxia may influence these dynamics and OAA can be redirected for aspartate rather than lactate synthesis. Moreover, mCAT expression appears to occur at higher levels than the expression of cCAT in colon cancer cell lines in both hypoxia and normoxia. Thus, mCAT supports cells according to the availability of O_2_ in the microenvironment, supplying both mitochondrial oxygen-dependent glutaminolysis and hypoxia-induced 2-oxoglutarate carboxylation [67].

Aspartate is essential for cancer cells to survive and proliferate, since it is the precursor in several pathways, including the synthesis of asparagine, as mentioned. Certain subsets of acute lymphoblastic leukemia cells are unable to synthesize asparagine and thrive on extracellular asparagine production to sustain proliferation and survival, thus being dependent on the functioning of CAT [66].

Cancer cells present increased glycolytic flux, aiming to obtain ATP and anabolic precursors. CAT functions as AST in MAS, allowing electron transfer from NADH through the mitochondrial electron transfer chain, intersecting with glycolysis, which suggests it can have important roles in the cancer context. However, the impact of AST on cancer cells still remains unclear [68]. Bayomi and colleagues reported that increased serum CAT levels correlate with cyclooxygenase 2 (COX-2)-encoding (*COX2*) mRNA expression in hepatocellular carcinoma [69]. COX-2 has pro-tumoral roles by inducing prostaglandin (PG) production, since it catalyzes the conversion of polyunsaturated fatty acids, usually arachidonic acid, to PGH_2_, which can then be converted into other PGs or tromboxanes [69,70]. This sustains angiogenesis through secretion of vascular endothelial growth factor (VEGF) [70,71]; acts as an ally in cancer cells apoptosis evasion through the release of anti-apoptosis factors; and supports cancer invasiveness. Thus, serum levels of CAT may be associated with COX-2 and this link may impact on hepatocellular carcinoma cancer cells and, possibly, on other types of cancer [71].

### 3.2. CAT as a Glutamate-Oxaloacetate Transaminase

**Figure 1:**
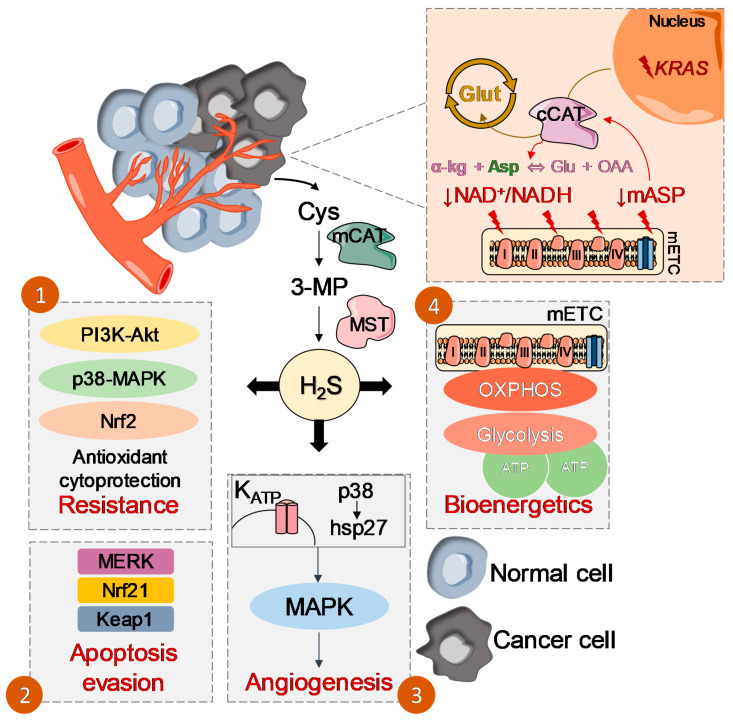
Role of the glutamate oxaloacetate transaminases: 3-mercaptopyruvate sulfurtransferase (CAT:MST) axis in promoting and sustaining cancer. Cancer cells presenting defective mitochondrial electron transport chain (mETC) exhibit low levels of NADH/NAD^+^ and mitochondrial aspartate (mAsp). These cells can take advantage of the reversible reaction catalyzed by cytoplasmic CAT (cCAT) (but not mitochondrial CAT (mCAT)), as observed in cancer cells with *KRAS* mutations, directing it to the production of Asp rather than its consumption, maintaining growth and survival. MST is pointed as a facilitator of cancer, probably due to multiple effects of H_2_S: (1) cytoprotection and chemoresistance by stimulating phosphoinositide 3-kinases (PI3K)—protein kinase B (Akt), p3—MAPK and nuclear factor erythroid 2—related factor 2 (Nrf2) pathways; (2) evasion of apoptosis by stimulating Nrf2, together with MERK and kelch-like ECH-associated protein 1 (Keap1); (3) angiogenesis promotion by increasing neovascularization through a K_ATP_ channel/p38/hsp27 pathway, leading to the activation of MAPK pathways, and (4) stimulation of cellular bioenergetics by directly injecting reducing equivalents into the mETC, and by persulfidation ATP synthase, glycolytic enzymes and lactate dehydrogenase.

Glutamate, which is generated from glutamine upon the action of glutaminase, can be metabolized in the mitochondria by mCAT at the expense of OAA, producing α-ketoglutarate and aspartate. Importantly, cCAT can also catabolize this reaction in the cytosol; however, α-ketoglutarate must then be transported across the mitochondrial inner membrane through a malate/α-ketoglutarate antiporter to enter the TCA cycle [72]. Glutamate represents the most profuse excitatory neurotransmitter in the vertebrate nervous system, enrolling in neurotoxic roles in certain neurological disorders or injuries [73]. Its metabolism in the brain through CAT was described by Krebs in 1935 [74]. Interestingly, CAT can induce the metabolism of otherwise neurotoxic extracellular glutamate by enabling its anaplerotic flux into a truncated TCA in stroke-injured brain cells, thus exerting a protective role upon cerebral ischemia [73,75]. Cancer cells presenting defective mETC can reversibly use cCAT but not mCAT as aspartate source to maintain its growth and survival. Defects in mETC lead to a decrease in the NAD^+^/NADH ratio and inhibit mitochondrial aspartate synthesis. However, given the bidirectional nature of the cCAT-catalyzed reaction (mentioned above), low levels of aspartate allow cCAT to partially compensate by reverting the flux towards aspartate production from OAA [64]. In addition to this, cancer cells presenting *KRAS* mutations also depend on cCAT to sustain their proliferation and survival by reprogramming glutamine metabolism, as described in pancreas cancer [76,77]. Highly proliferative cells can use glutamate derived from glutamine metabolism as a source of non-essential amino acids to sustain their proliferative rate. Thus, oncogenic *KRAS* promotes a shift in the cellular glutamine metabolism, relying on cCAT, as preferred by quiescent cells [51,76,77,78,79] (Figure 1). cCAT is reported as essential for maintaining redox homeostasis through the conversion of OAA into pyruvate in pancreatic ductal adenocarcinoma (PDAC) cells. cCAT depletion is associated with increased glucose consumption and lactate secretion levels, coordinating with glycolysis. cCAT thus arises as a vital enzyme for cell proliferation and cell viability in low-nutrient environments, possibly by its capacity to produce components to fuel the cancer remodeled metabolic networks, as OAA, and also as a way to regenerate NAD^+^ through the pyruvate-to-lactate conversion [80,81]. OAA is also a product of the degradation of malate by the malate dehydrogenase catalyzed reaction and is further acquired by cells through the activity of the ATP citrate lyase, through the cleavage of citrate. Interestingly, Zhou and colleagues have reported that supplementation of malate and succinate in *GOT1*-null cells did not rescue OAA levels, suggesting cCAT as the preferred way to acquire this intermediate, sustaining cell viability under starvation [80]. Moreover, cCAT may be linked to resistance to therapy in pancreatic cancer. Li et al. reported that *GOT1* suppression underlies radiosensitivity through increased ROS production induced by glutamine deprivation in pancreatic cancer stem cells [82]. In addition, mCAT was found to be critical in pancreatic cancer and it has been demonstrated that glutamine metabolism via mCAT induced premature PDAC cells senescence via the p27-dependent senescent pathway [83].

Regarding the response to oxidative stress, upon glutamine scarcity, the bidirectional glutamate-oxaloacetate transaminase activity of CAT also controls the de novo synthesis of GSH, since glutamate is a component of the GSH tripetide and it can be synthesized by CAT to sustain GSH levels [84].

### 3.3. CAT as a Cysteine Aminotransferase and as a Member of the CAT:MST Axis

**Figure 2:**
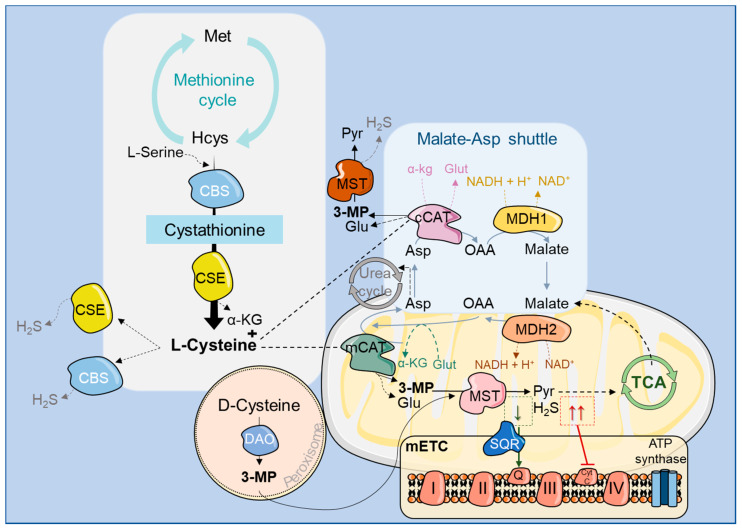
Metabolic pathways that rely on CAT activity. The methionine cycle produces homocysteine, which can be metabolized by cystathionine-β-synthase (CBS) and cystathionine-γ-lyase (CSE), two H_2_S synthesizing enzymes, leading to the production of l-cysteine. mCAT can then catalyze the transamination of l-cysteine, producing 3-mercaptopyruvate (3-MP) (and glutamate, Glu), which is then a substrate for MST, yielding H_2_S and pyruvate (Pyr), the latter entering the TCA cycle. d-cysteine can also be used in this reaction; however, it must be previously converted to 3-MP by the peroxisomal enzyme d-amino acid oxidase (DAO). At low concentrations, H_2_S stimulates the mitochondrial electron transport chain (mETC) by donating electrons that are transferred by sulfide:quinone oxidoreductase (SQR) to coenzyme Q (Q). At higher concentrations H_2_S binds to and inhibits cytochrome C oxidase (Cyt C), blocking electron transfer and energy production. CAT can also act on l-tyrosine, l-phenylalanine, l-tryptophan, and aspartate (Asp) metabolism, providing substrates that can enter other pathways, such as the TCA and the urea cycle. CAT is an essential element of the malate-aspartate shuttle that catalyzes the bidirectional conversion of aspartate (Asp) and α-ketoglutarate into oxaloacetate (OAA) and glutamate, with support from MDH1/2 enzymes.

The main role of CAT within the CAT:MST enzymatic system is to catalyze the production of 3-mercaptopytuvate and glutamate from α-ketoglutarate and l-cysteine (or d-cysteine derived from the peroxisomal enzyme d-amino acid oxidase, DAO). MST reacts with 3-mercaptopyruvate, resulting in the incorporation of a sulfane sulfur into the active site Cys248, thereby yielding a persulfidated MST, and releasing pyruvate [25,85,86]. The MST Cys248 persulfide (Cys-SSH) then reacts with a sulfane sulfur acceptor that can be a monothiol such as GSH, cysteine and homocysteine, or a dithiol, as dihydrolipoic acid and thioredoxin, the latter being the preferred and most efficient physiological acceptor. The persulfidated acceptor eventually releases H_2_S [13,34,36,87] (Figure 2).

**Figure 3:**
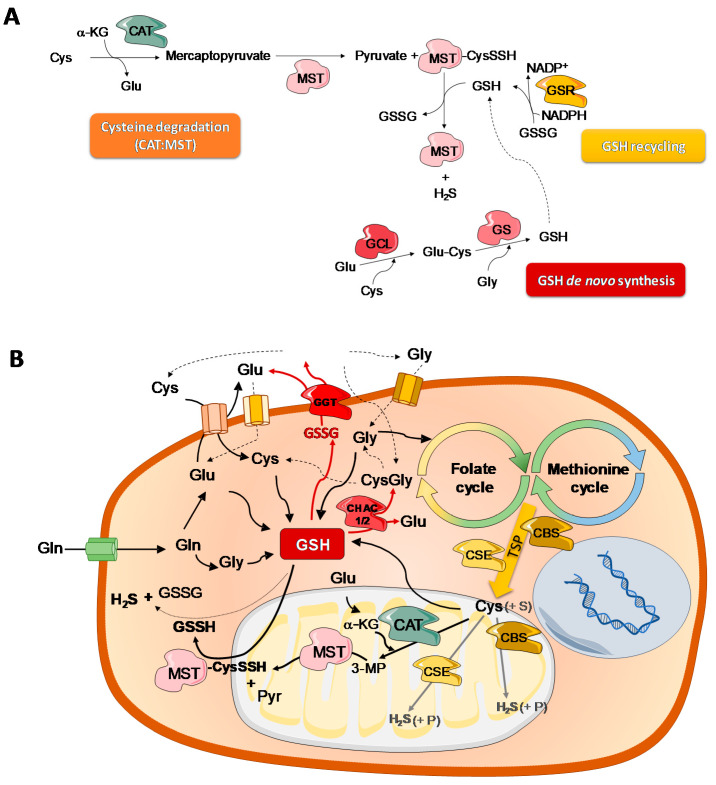
Interdependence of CAT:MST axis, glutathione (GSH) bioavailability and cysteine metabolism. (**A**) Glutathione (GSH) bioavailability depends on de novo synthesis and recycling. GSH is synthesized by the sequential action of two enzymes: Glutamate cysteine ligase (GCL), which links glutamate (Glu) to cysteine (Cys), and glutathione synthase (GS) that links the Glu-Cys dipeptide to glycine (Gly), forming GSH. Cysteine degradation by CAT:MST is dependent on GSH consumption, since CAT uses Cys and α-ketoglutarate (α-KG) to produce 3-mercaptopyruvate (3MP), which will be further used by MST to produce pyruvate (Pyr). In this reaction, MST becomes persulfidated (MST-CysSSH) and reacts with a thiol-containing or disulfide acceptor (e.g., GSH) that becomes persulfidated and ultimately releases hydrogen sulfide (H_2_S). (**B**) GSH synthesis depends on cysteine bioavailability, and Cys transport and metabolism depend on glutamine (Gln). Gln gives rise to Glu and Gly, two components of GSH. Glu is also needed for Cys uptake mediated by antiporters, while Gly supplies the folate cycle belonging to one-carbon metabolism together with the methionine cycle, from which homocysteine may originate Cys through the transsulfuration pathway (TSP), by the sequential action of cystathionine-β-synthase (CBS) and cystathionine-γ-lyase (CSE). Cys degradation and production of H_2_S by the CAT:MST axis requires Glu-derived α-KG and GSH. Cys can also be catabolized by CBS and CSE through a number of alternative reactions involving different substrate (S) combinations, releasing H_2_S and various products (P) (reviewed e.g., in [91]. GSH is a relevant source of Cys. The oxidized GSH (GSSG) export and degradation is mediated by γ-glutamyl transpeptidase (GGT) and it generates Glu and cysteinylglycine (CysGly) dipeptide, which will be further degraded, releasing Cys and Gly. The three amino acids are afterwards imported by specific transporters. GSH can also be degraded in the cytosol by glutathione-specific γ-glutamylcyclotransferase 1 and 2 (CHAC1 and CHAC2) isoenzymes, releasing Glu and Cys-Gly that will be again converted in Cys and Gly.

Therefore the release of H_2_S is highly dependent on GSH bioavailability, meaning that cysteine degradation by CAT:MST is regulated by the levels of GSH, whose synthesis in turn depends on cysteine bioavailability [88]. This interconnection is a way of controlling the levels of free cysteine in the cell, since it is thought to be mainly canalized to GSH or protein synthesis. However, cysteine degradation can be pivotal in cancer cell metabolic rewiring not only due to the production of H_2_S but also as an important carbon source, as recently reviewed by Serpa [89]. H_2_S is itself a powerful antioxidant [90], displaying a reduction potential very similar to the redox pair glutathione disulfide/glutathione (GSSG/GSH), the predominant scavenger of free radicals in mammals cells [91]. H_2_S controls oxidative stress in different ways: by stimulating the production of GSH through the activation of the expression of cystine/cysteine transporters, by directly interacting with ROS [92] and by modulating the persulfidation of different antioxidant response proteins, such as Keap1 [93] upstream activator of mitochondrial redox signaling p66Shc [94], and CuZn superoxide dismutase [95,96]. Besides this, at physiological levels, H_2_S has different roles in a cell. It can function as a gaseous signaling molecule that freely diffuses across aqueous and hydrophobic biological boundaries, being the third “gasotransmitter” in mammalians’ physiology, as extensively reviewed by Giuffré et al. [97]. Studies have shown that lower levels of H_2_S can act as an endogenous neuromodulator, inducing calcium release [98], and protect neurons from oxidative stress [99]; act as a smooth muscle relaxant [100] or as a vasodilator [101], as well as playing different roles in the inflammatory process [102]. Depending on its concentration, H_2_S may play a relevant role in bioenergetics. At low intracellular concentrations (0.01 to 1 μM), H_2_S may act as an electron donor to the mETC. H_2_S is oxidized by the membrane-attached mitochondrial sulfide:quinone oxidoreductase (SQR), which reduces coenzyme Q, while transferring a sulfane sulfur to an acceptor, such as sulfite or preferably GSH [97,103]. Reduced coenzyme Q is then reoxidized by complex III at the expense of cytochrome c reduction, which finally transfers electrons to cytochrome c oxidase to accomplish oxygen reduction [97], ultimately stimulating ATP production. However, at 3–30-fold higher concentrations, H_2_S reversibly inhibits cytochrome c oxidase with an inhibitory constant (*K*_i_) value of 0.2 μM at pH 7.4 [104,105] (Figure 2), which underlines the double-faced nature of H_2_S in cellular bioenergetics. Moreover, H_2_S mediates the persulfidation of glycolytic enzymes and lactate dehydrogenase, which also supports its regulatory role in the context of cancer, particularly colorectal [106]. Given this dual nature of H_2_S in mammalian physiology, imbalances in H_2_S levels have been correlated with various human pathologies, from cardiovascular and neurodegenerative diseases to cancer. Nonetheless, the abovementioned CAT’s substrate promiscuity can limit H_2_S production in an aspartate- and glutamate-dependent manner. Furthermore, CAT can additionally act on l-tyrosine, l-phenylalanine, and l-tryptophan metabolism, in the presence of α-ketoglutarate, leading to its catalysis plus the formation of glutamate [107], and in the urea cycle, by providing substrates for these pathways [108] (Figure 2). Moreover, the CAT commitment to cysteine degradation can be limited by the redox state of the cell (Figure 3A), since MST activity is conditioned by the levels of GSH. The GSH bioavailability is deeply influenced by oxidative stress that may increase cytoplasmic and mitochondrial NADP^+^/NADPH ratio, decreasing the glutathione reductase activity and driving GSH depletion that will lower the GSH/GSSG ratio and affect the cellular free radical scavenging capacity [109]. Certain cells, including cancer cells, ensure the antioxidant capacity renewal by increasing GSH de novo synthesis instead of investing in the GSH recycling [3,110].

Importantly, GSH catabolism is a main source of cysteine, together with cysteine anabolism through the transsulfuration pathway (TSP). GSH can be degraded outside or inside of the cell, being predominantly the extracellular catabolism of GSH a source of cysteine [111]. The degradation of oxidized glutathione (GSSG) through the γ-glutamyl cycle (Figure 3B) will allow recycling of glutamate, cysteine and glycine outside of the cell. These amino acids are subsequently imported by specific transporters. Upon export, GSSG is degraded by enzymes located at the external layer of the cell membrane. The first enzyme, γ-glutamyl transpeptidase (GGT) generates glutamate [112] and the cysteinylglycine dipeptide, which will be further degraded by dipeptidases such as aminopeptidase N (APN), releasing cysteine and glycine [113]. The cysteinylglycine dipeptide can also be imported by cells using the peptide transporter 2 (PEPT2), undergoing degradation in the cytoplasm by unspecific dipeptidases [114]. The intracellular GSH catabolism is catalyzed by glutathione-specific γ-glutamylcyclotransferase 1 and 2 (CHAC1 and CHAC2) isoenzymes [115,116], releasing cysteinylglycine in the cytosol. Despite little being known about these enzymes in cancer, the CHAC2 isoform has been classified as a tumor suppressor gene in gastric and colorectal cancer studies, correlating CHAC2 downregulation with more aggressive cancer phenotypes and its activation with cancer cells poor survival [117].

cCAT and mCAT intersect with the methionine cycle through the ability of both enzymes to use cysteine and its sulfinate derivative as substrates, releasing glutamate and leading to the production of pyruvate. Homocysteine derived from the methionine cycle is converted through the transsulfuration branch, involving cystathionine β-synthase and cystathionine γ-lyase, to l-cysteine. Furthermore, the latter can be oxidized to l-cysteine sulfinate by cysteine dioxygenase.

The CAT:MST axis is particularly associated with critical and related physiological functions, as detoxification, transsulfuration, and H_2_S and per- and poly-sulfides production. CAT and MST expression was reported in endothelial cells of the thoracic aorta, indicating that vascular endothelium produces H_2_S through the activity of this enzymatic system, allowing H_2_S to act as a smooth muscle relaxant released from endothelium [34,118]. Moreover, a role of MST in anti-oxidative stress and redox sensing was also described, contributing for the cellular redox homeostasis [119,120]. Moreover, MST seems to play a critical role in the central nervous system. Indeed, MST knockout mice presented anxiety-related behaviors, which could be related to the lack of oxidative stress scavenging due to MST absence [121]. Physiological functions of rhodanese-like proteins, like MST, are further associated to the homeostasis of cellular sulfur in general. Furthermore, MST is involved in the biosynthesis of enzymatic cofactors, vitamins, and sulfur-containing nucleic acids [37]. The relevant role of MST in human physiology is highlighted in individuals with mercaptolactate-cysteine disulfiduria (MCDU) due to *MPST* mutations, who present a deficient methionine cycle and sulfur amino acid metabolism in erythrocytes, and excrete elevated levels of mercaptolactate-cysteine disulfide in the urine [122,123,124]. MST deficiency leads to a shift in mercaptopyruvate degradation towards lactate dehydrogenase, producing mercaptolactate [35]. MCDU is a rare and inheritable disorder that is thought to be linked to mental retardation in some patients [121]. This clinical evidence may be associated to the impaired production of antioxidant agents during the brain embryonic development. However, the mechanism through which MST deficiency promotes mental retardation is not yet elucidated [35].

## 4. Role of the CAT:MST Axis in Cancer Metabolic Remodeling

Increasing evidence supports the CAT:MST axis as displaying crucial roles on cancer metabolic rewiring. Besides the altered expression of mCAT and cCAT in cancer, increased expression of *MPST,* the gene encoding for MST, was found in several types of cancer, including liver, colorectal, endometrial, stomach, prostate, urothelial, and pancreatic carcinomas [41].

Regarding MST enzyme expression and activity, albeit MST is constitutively expressed in normal differentiated cells, some studies also detected its expression or activity in several different cancer cell lines and primary tumors, including brain, colon, liver, kidney, lung, bladder, and melanoma (reviewed by Augsburger and Szabo) [125]. In some of those studies, MST expression was significantly augmented [126,127], and an association between MST expression and chemoresistance was described in colorectal, liver and breast carcinoma [106,128]. Nevertheless, few functional assays tried to correlate the expression and/or activity of MST with cancer cells features. Studies testing MST inhibition or silencing showed that MST activity is important for colon and lung cancer cells proliferation [129,130]. This suggests a critical role of CAT and MST enzymes expression and activity in cancer, with increased importance regarding the associated regulatory mechanisms, according to cancer type and, probably, the metabolic context. Although the role of MST in cancer is evident in the control of metabolic reliance, cell survival, and proliferation, *MPST* expression regulation upon carcinogenesis and its fitness into the progressive cancer phenotype is not often explored.

The exact role of CAT:MST in cancer development and progression remains under investigation, but several studies have focused on its final reaction product, H_2_S, and its role on cancer cells functioning. In fact, several cancer cell lines and tumor specimens have been shown to overexpress one or more of the H_2_S-synthesizing enzymes, including MST, resulting in increased H_2_S levels, which have been proposed to promote carcinogenesis through the regulation of various cancer-related processes [97]. In the last decade, a novel concept emerged in the field of cancer biology, demonstrating that various cancer cells can increase their endogenous H_2_S levels and use it in an autocrine and paracrine manner to promote cell proliferation, cytoprotective signaling, angiogenesis, and stimulate cellular bioenergetics [131]. In fact, several biological roles regarding H_2_S in cancer cells have been proposed [131], which reinforces the importance of the CAT:MST system in cancer context. For instance, MST was found to be upregulated in multidrug-resistant and stem cell-like cancer cell lines when recovered from stressful or cytotoxic stimuli [106,128], suggesting a possible cytoprotective role of MST in drug-resistant and advanced cancers [125]. H_2_S is indeed described as an angiogenesis promoter by positively influencing endothelial migration in vitro [132,133]. H_2_S can be associated to increased neovascularization relying on a K_ATP_ channel/p38/hsp27 pathway, acting through the facilitated activation of mitogen-activated protein kinases (MAPK) pathway by K_ATP_ channels. H_2_S has an inhibitory effect on cGMP phosphodiesterases. Thus, H_2_S ultimately leads to new blood vessel formation and vasodilation [132,133]. Physiological levels of H_2_S are further associated to increasing levels of mitochondrial cAMP [105,134] by mediating the persulfidation of mitochondrial ATP synthase and lactate dehydrogenase A [135,136], endogenously stimulating cellular bioenergetics [105]. In addition, H_2_S promotes antioxidant cytoprotective effects by stimulating PI3K–Akt, MAPK, and Nrf2 pathways [137,138,139,140] (Figure 1). The influence of H_2_S on cancer cell proliferation, migration, invasion, and resistance was further confirmed in colon [141] and ovarian cancer [142]. Several studies suggested that MST silencing, which consequently impedes the catabolism of products directly derived from CAT enzymatic activity, suppresses cancer cell bioenergetics and/or proliferation in hepatoma and lung carcinoma cell lines [125,143]. MST was found to be upregulated in 5-fluorouracil (5-FU)-resistant colonic HCT116 cancer cells, concomitant with an increased capacity to synthesize H_2_S [106], thus suggesting that increased H_2_S synthesis in cancerous colon cells may increase their capacity to cope with chemotherapeutic drugs [144]. In lung adenocarcinoma cells, increased H_2_S production was associated with the boosting of the mitochondrial DNA (mtDNA) repair mechanism and with bioenergetics flux sustenance. Abrogation of the H_2_S-enhanced production induced mitochondrial dysfunction due to the accumulation of mutations in mtDNA, sensitizing tumor cells [130]. mtDNA is in fact a major source of cancer cells vulnerability and, when damaged, a cascade of events, including activation of mitochondrial cell death pathways, compromised regulation of mitochondrial protein expression and imbalances in cell bioenergetics’ homeostasis, leads to cancer cells’ fatality [145]. Szczesny et al. showed that inhibiting the H_2_S synthesizing enzymes and, consequently, H_2_S production, results in accumulated DNA repair intermediates in mtDNA, which possibly occurs due to H_2_S role in the stabilization of mtDNA repair machinery being, thus, essential for the well-functioning of those complexes [130].

It should be highlighted that not only cysteine degradation with H_2_S production but also the cysteine degradation blockade and its diversion to GSH synthesis, account for chemoresistance, since GSH is able to block the mechanism of action of drugs commonly used to treat cancer [146,147,148]. Thus, cysteine catabolic systems, such as the CAT:MST axis, may have a role in this induced chemoresistance. Cancer cells present high levels of GSH, which is a determinant ally for cancer cells survival upon chemotherapy, by directly reacting with drugs and generating adducts for excretion, abrogating ROS and consequently preventing protein and DNA damage and ultimately cell death [149]. Accordingly, Nunes et al., showed that cysteine promotes chemoresistance of ovarian cancer cells in a hypoxic environment, which is probably related to its known antioxidant effect, leading to cancer progression [147]. High cysteine levels were further found in ascitic fluid and in serum from ovarian cancer patients, which indicates that cysteine metabolism can promote the adaptation of neoplastic cells against hypoxia, having a role as a redox buffer, and against chemotherapy, as sulfur rapidly binds to platinum-based cancer chemotherapy, preventing drug efficacy [147].

Hypoxic conditions are common in TME, which may trigger cancer cells to develop protection mechanisms from the reduced oxygen levels and, probably, to reprogram their metabolism towards oxygen non-requiring energy production ways. In fact, a high rate of glycolysis followed by lactic acid production, the Warburg effect, is a common feature of cancer cells. However, cancer cells do not completely abrogate OXPHOS [1,150,151,152,153], but rather fulfil OXPHOS mainly using other substrates besides glucose, such as lactate, fatty acids and glutamine [154]. Therefore, the increased rate of glycolysis in cancer aims to supply other glucose dependent pathways to sustain biomass production and cell proliferation, as the pentose phosphate pathway [154,155]. Importantly, in addition to ATP production, H_2_S has also been implicated in the regulation of glycolysis, which is particularly relevant in highly proliferating cancer cells. Furthermore, an inorganic source of H_2_S (NaSH) was shown to enhance glucose uptake and glycolysis efficiency in cardiomyocytes [156] likely both by stimulating the activity of glucose transporter and of glyceraldehyde 3-phosphate dehydrogenase (GAPDH) [157,158], a finding that was further reported in a panel of cancer cell lines [159].

More studies addressing the particular role of CAT:MST enzymatic system in the ability of cancer cells to reprogram their metabolism are essential to understand and establish cancer metabolic patterns that could be useful in cancer diagnosis/prognosis and also in personalized medicine, since the linkage between TME and cancer cells may be a key factor in determining the course of cysteine flux within cancer cells [160]. Moreover, cysteine metabolism in tumors may set the route for patient stratification according to individual metabolic features, allowing to redirect patients who will probably not benefit from chemotherapy to alternative therapies. Given the critical role of H_2_S-producing systems like CAT:MST in cancer, their pathophysiological activity may contribute for cancer metabolic remodeling, supplying the rewired metabolic pathways in cancer cells, ultimately allowing their survival and resistance to therapy.

## 5. Targeting CAT:MST Axis to Treat Cancer

CAT enzymes have a transversal role in the overall metabolic network depending on amino acids metabolism; thus, the inhibitory effect of CAT has been explored in different models and mostly in an MST-independent perspective.

Looking at the metabolic perspective, cCAT depletion induces increased aspartate levels that can be directed to nucleotide synthesis [64,76]. Aspartate is normally synthesized in the mitochondria by the sequential actions of MDH2 and mCAT and is then carried to the cytosol to serve as a substrate for cCAT and other enzymes [64], as mentioned, implying that cCAT and mCAT rely on each other and that inhibition of one form of CAT may impact on the activity of the other. Indeed, Chen et al. reported that mCAT attenuation alone can impact on nucleotide synthesis [161]. Hypoxia-inducible factor 1 alpha (HIF1α) was found to simultaneously inhibit cCAT and mCAT, limiting aspartate biosynthesis through a combined repression of MAS [162]. Several studies reported inhibitors towards CAT, particularly the cCAT form, which led to anti-tumoral effects. In pancreatic cancer, Yoshida and colleagues tested an inhibitory compound (PF-04859989) that covalently bonded to cCAT, promoting inhibitory effects in a time- and PLP-dependent fashion, further showing selective anti-proliferative effects towards pancreas cancer cell lines [163]. Sun et al. reported the tumor suppressive effects of Aspulvinone O (AO), a natural compound isolated from *Aspergillus terreus* and found it to be a selective inhibitor for cCAT in PDAC cells but not healthy cells, both in vitro and in vivo. AO induced cell cycle arrest, triggered apoptosis, and decreased the colony formation ability of pancreatic cancer cells, leading to decreased tumor size in a SW1990 cell-induced xenograft model. AO was further reported to competitively bind to and form a complex with cCAT, inhibiting its intracellular activity and, thus, targeting glutamine metabolism activity in pancreatic cancer cells [164], essential to sustain their proliferation and support tumor growth, as mentioned above [76]. In ovarian cancer, Wang et al. reported that adapalene (ADA), a third-generation retinoid used in clinical practice for the treatment of *acne vulgaris,* had inhibitory effects on cCAT in vitro. ADA inhibited cell migration and stimulated apoptosis on ES-2 cells. Importantly, ADA showed to be more effective on cancer cells overexpressing cCAT [165]. A study on acute myeloid leukemia cells revealed that mCAT disruption inhibited cell proliferation [161].

The CAT:MST axis has critical importance for the functioning of physiological metabolic processes, but it has also a crucial role in cancer, particularly in cancer metabolic remodeling. Overexpression of CAT and MST is associated with protective effects on cancer cells, accounting for therapy resistance, hence strategies that abrogate this overexpression can chemo- and radio-sensitize cancer cells [106,166,167]. As mentioned, cCAT has been more extensively studied as a therapeutic target in cancer than MST. However, given their overall crosstalk and interdependence, CAT inhibitors are expected to indirectly impact on MST activity due to shortage of 3-MP substrate. This is supported, for example, by the non-selective PLP-inhibitor aminooxyacetic acid, that impairs H_2_S production by MST through inhibition of CAT [168]. Furthermore, most studies focused on unravelling the effects of inhibitors targeting other enzymes in cysteine catabolism and H_2_S production, rather than MST [168].

Several compounds with inhibitory effects on MST activity are known, as the chemical compounds hypotaurine and methanesulfinic acid, and the substrate-like inhibitors pyruvate (its product), phenylpyruvate, oxobutyrate, oxoglutamate, among others [168,169,170,171]. However, these compounds present both minimal efficacy and lack of selectivity [169,170,172], which makes them scientifically unattractive to pursue biologic studies [168].

Hanaoka et al. recently identified through a high-throughput screen a highly effective and selective rat MST inhibitor that in theory can interact with the persulfidated cysteine in the active site of MST, without interfering with the other H_2_S producing enzymes [172]. Although studies are lacking regarding the effect of these inhibitors in cancer, there is the possibility that an MST inhibitor targeting the persulfidated cysteine active site affects off-target mitochondrial and cytoplasmic proteins that are prone to persulfidation. [125]. It is particularly interesting that the cancer cells ability to recover from cell injury is correlated with increased MST expression [106,128], which indicates that effective MST inhibitors may have a strong impact on cancer cells survival, being a valuable therapeutic weapon.

## 6. Concluding Remarks

Metabolism reprogramming is an established hallmark of cancer. Several pathways which cancer cells rely on for invasive and proliferative potential have been dissected over the years, aiming to understand the metabolic puzzle that is certainly an important cue in cancer and that can lead to improvements in current diagnosis and prognosis tools and therapeutic strategies.

Amino acids metabolism is a crucial core in the entire cellular metabolism and here we showed that CAT plays a relevant role in the metabolic network by catalyzing the interconversion of different amino acids (Figure 3B). Glutamate and cysteine are two components of GSH, and CAT uses both these amino acids as substrate. Thus, their bioavailability, directly dependent on CAT, will condition the antioxidant capacity of cancer cells mediated by GSH. This is a tricky point in the oxidative stress control, since GSH is the main non-protein cellular scavenger and its depletion leads the cell to undergo ferroptosis and death. In the other hand, the degradation of cysteine by CAT:MST promotes H_2_S production, which presents antioxidant properties and can somehow overcome the depletion of GSH in oxidative stress control. Moreover, cysteine is placed as a relevant carbon source, since pyruvate is a product from its degradation by CAT:MST. Glutamine, besides controlling GSH synthesis by originating glutamate and glycine, also acts on the control of cysteine metabolism, degradation, and de novo synthesis (Figure 3B). By originating glutamate and sequentially α-ketoglutarate, glutamine controls the degradation of cysteine through CAT:MST axis; and by originating glycine to supply the one carbon metabolism, glutamine controls the cysteine synthesis through the transulfuration pathway.

The CAT:MST enzymatic axis is an important system with growing interest in cancer metabolism, since it is known to be altered in cancer with an established association to cancer progression and poor prognosis. Nevertheless, amongst the cysteine degradation/H_2_S production pathways, the CAT:MST pathway is under explored. Its pro-cancer role is related to the production of essential molecules and energy to answer cancer cells demands. However, deeper insights are necessary to uncover the crosslinks established within the CAT:MST axis metabolism in cancer cells and to understand if the accumulation of antioxidant agents that induce chemoresistance can be affected by a balance between different pathways and molecules, in particular the amino acids whose fate is controlled by CAT. Perhaps the most relevant aspect of CAT in cancer metabolism is the fact that it sits precisely at the crossroads of amino acids metabolism, antioxidant response capacity and H_2_S-based signaling, all crucial factors for cancer cell adaptation within the tumor microenvironment. Therefore, the CAT:MST axis encloses a high potential of therapeutic management, mainly by targeting CAT which should disrupt and disturb the pathophysiological homeostasis sustaining cancer cells survival. Thereby, it will help in the definition of new therapeutic strategies and drugs, accounting for the improvement of the oncological disease management.
